# Analysis of Mechanical and Wettability Properties of Natural Fiber-Reinforced Epoxy Hybrid Composites

**DOI:** 10.3390/polym12122827

**Published:** 2020-11-27

**Authors:** Ayyappa Atmakuri, Arvydas Palevicius, Madhusudan Siddabathula, Andrius Vilkauskas, Giedrius Janusas

**Affiliations:** 1 Faculty of Mechanical Engineering and Design, Kaunas University of Technology, Studuntu 56, 44249 Kaunas, Lithuania; arvydas.palevicius@ktu.lt (A.P.); andrius.vilkauskas@ktu.lt (A.V.); giedrius.janusas@ktu.lt (G.J.); 2 Faculty of Mechanical Engineering and Design, Usha Rama College of Engineering, Telaprolu, Andhra Pradesh 521109, India; madhusudan_sunanda@rediffmail.com

**Keywords:** natural fibers, hemp and flax fibers, banana and pineapple fibers, hybrid composites, mechanical properties, contact angle, moisture absorption

## Abstract

Natural fibers have many advantages over synthetic fibers due to their lightness, low cost, biodegradability, and abundance in nature. The demand for natural fiber hybrid composites in various applications has increased recently, because of its promising mechanical properties. In this research work, the mechanical and wettability properties of reinforced natural fiber epoxy resin hybrid composites were investigated. The main aim of this research work is the fabrication of hybrid composites and exploit its importance over individual fiber composites. The composites were fabricated based on the rule of hybridization mixture (0.4 wf) of two fibers using sets of either hemp and flax or banana and pineapple, each set with 40 wt%, as well as four single fiber composites, 40 wt% each, as reinforcement and epoxy resin as matrix material. A total of two sets (hemp/flax and banana/pineapple) of hybrid composites were fabricated by using a hand layup technique. One set as 40H/0F, 25H/15F, 20H/20F, 15H/25F, 0H/40F, and the second one as 40B/0P, 25B/15P, 20B/20P, 15B/25P, 0B/40P weight fraction ratios. The fabricated composites were allowed for testing to examine its mechanical, wettability, and moisture properties. It has been observed that, in both cases, hybrid composites showed improved mechanical properties when compared to the individual fiber composites. The wettability test was carried out by using the contact angle measurement technique. All composites in both cases, hybrid or single showed contact angle less than 90°, which is associated with the composite hydrophilic surface properties. The moisture analysis stated that all the composites responded for moisture absorption up to 96 h and then remained constant in both cases. Hybrid composites absorbed less moisture than individual fiber composites.

## 1. Introduction

Recently, many researchers have become interested in working with natural fiber hybrid composites, because of its abundance, light weight, low cost, high specific properties, and the fact it does not pose any harm to the environment. Natural fibers are finding increasing use as reinforcement materials in polymer matrix composites, often replacing synthetic (manmade) fibers [1]. Hybrid fiber composites are materials that consist of two or more reinforcements and the same matrix material, and the procedure is called a hybridization process [2]. The matrix serves as binding material and gives the extra strength to the composite [3]. Composites are classified as per matrix and reinforcement materials. Based on the matrix material, composites are classified as metal matrix composites (MMCs), ceramic based composites (CMCs), and organic matrix composites (OMCs), based on reinforcement material composites are classified as laminar composites, particulate composites, and fiber reinforced composites (FRCs) [4,5,6]. In the fiber reinforced composites, fiber material serves as reinforcement material and works as a stiffness agent. The matrix material does not only serve as a binding agent but also distributes the external forces (load) acting on the composite to the fibers. The majority of the external load is sustained by the reinforcement materials and a small portion is carried by the matrix material.

The fibers are classified as natural fibers and synthetic fibers. Abaca, bamboo, banana, cotton, coir, flax, hemp, jute, kenaf, pineapple, and sisal are some examples of natural fibers. Boron, carbon, ceramic, and glass are some examples of synthetic fibers. They are also called manmade fibers. Synthetic fiber composites have so many drawbacks when compared to natural fiber composites, such as rapid burning, not being good for the environment, not being skin-friendly and therefore uncomfortable for extensive wear [7]. Whereas natural fibers are biodegradable, low cost, environmentally friendly, light in weight, have great availability in nature, and are renewable. The biodegradability of natural fibers shows a major impact in balancing a healthy environment with inexpensiveness and high specific performance, which attract the economic interest of many industries. The physical and mechanical properties of natural fibers and their composites are still being explored.

Hybrid composites are fabricated to maintain the advantages of its constituents and overcome some of the drawbacks. The concept of hybridization is successful approach in plant fibers to address the deficiency of ductility of the composites [8]. Hybridization is defined as when the composites are fabricated with two or more reinforcements and are joined under the same matrix (resin) material with 0.4 wf ratio. As reported by many researchers, the hybridization of different fibers under varying weight fractions tends to a gradual increase in mechanical, physical properties, and also cost-effective composite materials. The main advantage is that the mixing of the fibers can be done with various methods, such as intermixed continuous fibers, intermixed discontinuous fibers, intermingled particulate fibers, aligned short fibers, and sandwich layers [9,10]. Due to the hybridization of natural fibers, composites showed greatly enhanced mechanical properties. A recent survey stated that the fabrication of natural fiber reinforced polymer composites has become more popular in commercial applications, such as in the construction, automobile, military goods, sports equipment, aerospace, and shipping industries. Fernanda Luz et al. [11] worked on composites with natural fibers and conventional materials applied in a hard armor. The results stated that the usage of natural fiber composites in armor plates to reduce the thickness of the hard armor plate. The experimental results stated that the natural fiber-reinforced composites have the potentiality to replace ceramic-based composites. Neuba et al. [12] investigated the mechanical, thermal, and ballistic properties of novel epoxy composites reinforced with Cyperus malaccensis sedge fiber. The results indicated an improvement in mechanical properties when compared to the neat epoxy composites. Yorseng et al. [13] worked on weathering studies of kenaf and sisal fiber reinforced hybrid composites for semi-structural applications. The mechanical and thermal properties of natural fiber hybrid composites have more enhanced properties than pure epoxy composites at both before and after weathering conditions. From the results, it is a considerable advantage to use bio-based composites in semi-structural applications.

In general, the chemical composition plays an important role in the natural fibers and it differs from fiber to fiber. The overall performance is mostly reliant on the aspect/ratio and cellulose crystallinity of the fiber materials. Cellulose plays a prominent role in the plant fibers, and it shows an adverse effect on moisture absorption. Hemicellulose and lignin are also major components, in addition to by cellulose. Cellulose occupies the major place in the plant fibers, and it comprises up to 55% of the total. Lignin serves as cementing material and its stimulus the surface morphology and properties [14]. Hybrid composites made from plant fibers and their by-products are the most widely used lignocellulosic materials, due to their enhanced mechanical properties. Banana and pineapple are the most cultivated plants, providing leaf fibers for composite applications in India. These leaf fiber composites and their by-products utilization increased in a wide range of commercial applications. Bast fiber composites, such as leaf fibers, especially hemp and flax fiber composites, are also becoming popular. Hemp and flax fibers are mostly cultivated in European countries [15]. Researchers are showing much interest, due to its mechanical performance and lightweight applications. In agreement with the European Commission, the use of waste raw materials obtained from the processing of bast fibers, such as hemp and flax, is an ailment for the balanced use of plants [16]. Along with reinforcement material, matrix material also plays an important role in the hybrid composites. The structure and surface morphology properties are based on resin material. Based on dispensation techniques, matrix materials are classified as thermosetting and thermoplastic materials. A matrix material works as a load distributor in a composite when an external force acts on it [17]. Thermosets are widely popular for their exclusive property of forming 3D bonds after curing the composite. They are a contrast to plastic polymer materials, and have great mechanical performance, thermal stability, and resistance to creep [18]. Thermoset polymers are a more widely used resin material for composite fabrication than thermoplastic polymers. Thermosetting materials have superior mechanical properties, thermal stability, and ease of processing. Thermosets are easy to process and do not require additional heat treatment process for composite curing. Unlike thermoplastic material, these are not recyclable. Polystyrene, polyethylene, polyvinyl chloride, and natural rubber are some of the examples for thermoplastic resins. Epoxy, phenolic, polyester, vinyl ester, urea-formaldehyde, and unsaturated polyester are some examples of thermoset resins. Researchers are showing much interest in using epoxy resin, due to its exponential mechanical properties, low curing time, durability, availability, and low cost [19]. Epoxy resin—along with hardener used as a matrix material in the current research.

Many researchers found that reinforcing with natural fiber with epoxy resin matrix has given enhanced properties. Sapuan et al. [20] worked on the mechanical properties of banana fiber reinforced epoxy resin composites. The results stated that banana fiber composites showed improvement in flexural strength, durability, and stiffness. Al Rashid et al. [21] studied the utilization of banana fiber reinforced hybrid composites in the sports industry. Due to its extensive mechanical properties, banana fiber composites are a considerable replacement for glass fiber composites in the sports industry. Karimzadeh et al. [22] studied the effect of stacking sequence on the mechanical properties and moisture absorption characteristics of hybrid pineapple/glass fiber composites. The results indicated that hybrid composites have superior flexural strength, tensile strength, and tensile strength than pure composites. Pure pineapple absorbed more percentage of water than hybrid composites. Shivamurthy et al. [23] investigated the sliding wear, moisture absorption, flammability, and mechanical properties of banana short fiber/Al(OH)_3_ epoxy composites. The mechanical properties, such as tensile strength and hardness, improved significantly due to the addition of natural fibers, and these properties are enhanced by increasing the fiber material to a certain extent. Neves et al. [24] worked on comparative mechanical properties between bio composites of epoxy and polyester matrices reinforced by hemp fiber. Hemp fiber composites with epoxy resin showed greater properties than polyester composites. Epoxy and polyester resin composites showed flexural strength as 66.7 and 49.1 MPa, and tensile strength as 50.5 and 25.4 MPa, respectively. Thiagamani et al. investigated the mechanical, moisture absorption, and swelling behavior of hemp/sisal fiber reinforced bio-epoxy hybrid composites: effects of stacking sequences [25]. The experimental results stated that hybrid composites displayed small variation in tensile strength when the stacking pattern was changed. Hybrid composites showed 40% higher mechanical properties than individual fiber composites. Moudood et al. [26] studied the mechanical properties of flax fiber reinforced composites at different relative humidifies: experimental, geometric, and displacement potential function approaches. The experimental results indicated that improved tensile strength and tensile strain in natural hybrid fiber composites. Mohammed Sh. Al-Otaibi et al. [27] worked on the characterization of date palm fiber-reinforced different polypropylene matrices. In this study, the authors investigated the mechanical, morphological, and thermal properties of date palm fiber composites. It was found that the reduction in tensile properties and improvement in modulus, when compared to the neat resin composites and date palm fiber composites, show advanced thermal and morphological properties. Junio et al. [28] worked on Copernicia prunifera leaf fiber epoxy composites. The results showed a 40% increase in tensile properties and a 69% increase of the elastic modulus, when compared to the neat epoxy resin composites.

In this research work, two sets of hybrid composites (five different weight fractions) based on epoxy resin and reinforcement with four types of natural fibers, hemp (H), flax (F), banana (B), and pineapple (P), were fabricated by using hand lay-up technique. The fabricated composites were allowed for material properties testing, such as flexural strength, flexural modulus, interlaminar shear strength, contact angle measurement, and moisture absorption. The mechanical performance of the two sets of fabricated laminates was investigated and the results were compared.

## 2. Materials and Methods

### 2.1. Materials

The reinforcement materials used for the preparation of samples are banana, flax, hemp, and pineapple natural fibers and epoxy resin. The mechanical properties and chemical compositions of these fibers are given in Table 1. Banana and pineapple fibers are a type of leaf fiber that is readily available in nature. These are widely available in India. Whereas hemp and flax fibers are a type of bast fiber, which is extracted from the banks of the plants. Epoxy resin, along with hardener, was used as a matrix material and it was purchased from Composites 24, Riga, Latvia. The mold material was made from teak wood.

### 2.2. Preparation of Fibers

**Figure 1:**
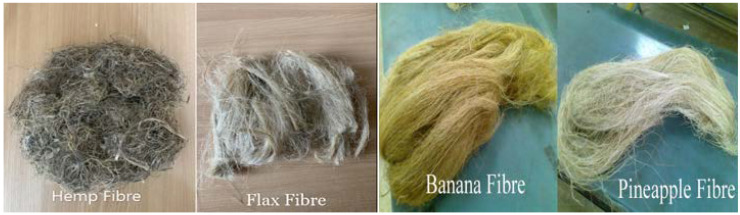
Fibers used for the fabrication.

Fiber selection and extraction is one of the major parts of the research. All the obtained fibers were cleaned with water and then dried to eliminate the water in it. Then, the fibers were segregated slightly with hand sitting patiently. The fiber laminates were allowed for the hand retting process to separate individual fiber strands by using a mechanical combing process. After separating them, fibers were looked over with a cotton checking outline several times, to isolate the filaments. Then, fibers were allowed to dry at room temperature to eliminate any moisture. After that, all the fibers are measured for weight and length, as per the required dimensions. The fibers used for the fabrication purpose are shown in Figure 1.

### 2.3. Weight Fraction of Reinforcement and Matrix Materials

The weight fraction of the constituents used for the fabrication process was considered based on the hybridization process. In the present work, total two sets of banana(B)/pineapple(P) and hemp(H)/flax(F) composites were fabricated by varying the weight fraction of fibers, such as 40B/0P (40% banana fibers), 25B/15P (25% banana and 15% pineapple), 20B/20P (20% banana and 20% pineapple), 15B/25P (15% banana and 25% pineapple), 0B/40P (40% pineapple fibers), 40H/0F (40% hemp fibers), 25H/15F (25% hemp and 15% flax), 20H/20F (20% hemp and 20% flax), 15H/25F (15% hemp and 25% flax), and 0H/40F (40% flax fibers).

### 2.4. Preparation of Matrix Material

Epoxy resin, along with hardener, was used as a matrix material. For the proper resin solution, the weight proportions of both resin and hardener were considered as 10:2 ratio as per the instructions. The epoxy resin and hardener were taken into a plastic container and mixed for 2–3 min at room temperature with a plastic stirrer, until the mixture was uniform in color. Then, the solution was stirred for another 30 s to scrape the side and bottom of the container. After sufficient mixing of the hardener and epoxy, the resin solution was applied to fibers. The mechanical properties of epoxy resin and hardener are in the following Table 2.

### 2.5. Preparation of Mould

**Figure 2:**
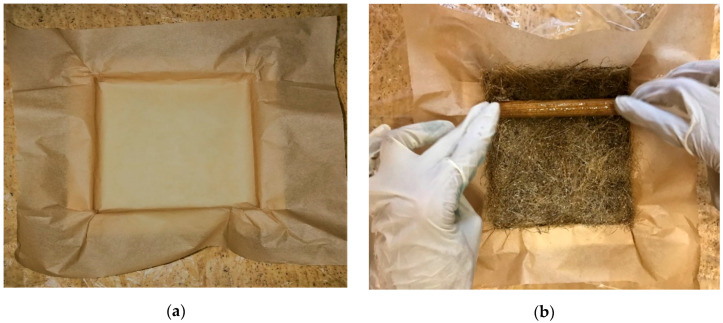
Mold material for fabrication purpose (**a**); roller on the composite surface (**b**).

Mold material used in this work was made from teak wood, as shown in Figure 2a. The dimensions of the mold are 150 mm × 150 mm × 12 mm. In general, matrix material sticks to the surface of the mold material. To avoid sticky nature, the mold is covered with baking paper, because it does not show any impact on mechanical properties. After placing the fibers inside the mold resin material applied to it and a roller was used, as shown in Figure 2b, to get the uniformity.

### 2.6. Composite Fabrication

**Figure 3:**
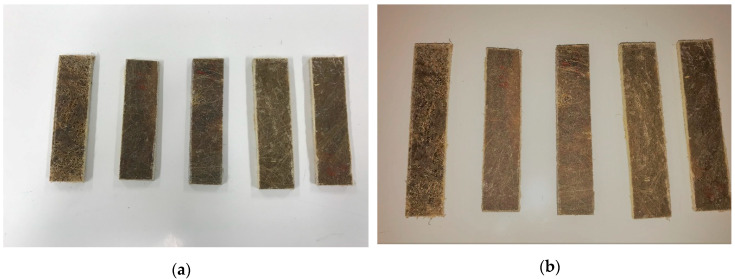
Hemp and flax fiber hybrid composites (**a**); banana and pineapple fiber hybrid composites (**b**).

Banana/pineapple, hemp/flax fiber hybrid composites were fabricated by using hand lay-up techniques. The mold materials were cleaned properly before placing the fibers in it. The mold was covered with baking paper and the fibers were placed in the form of beds, before applying the resin. After pouring epoxy resin in the mold material, it was compressed for a few minutes with a roller to spread the epoxy in all the corners of the mold. This avoids the gaps and formation of holes in the composite. A uniform load of 6KN was placed on the top of the composite surface and allowed for a curing time of 24–28 h. For each weight fraction, a total of five samples were prepared, to take the average values and ensure the specimens are cut as per ASTM standards. The fabricated samples of hemp/flax and banana/pineapple hybrid composites are shown in Figure 3a,b. The weight fractions of the composites are 40B/0P, 25B/15P, 20B/20P, 15B/25P, 0B/40P, 40H/0F, 25H/15F, 20H/20F, 15H/25F, and 0H/40F.

## 3. Testing of Composites

### 3.1. Flexural Strength Test

**Figure 4:**
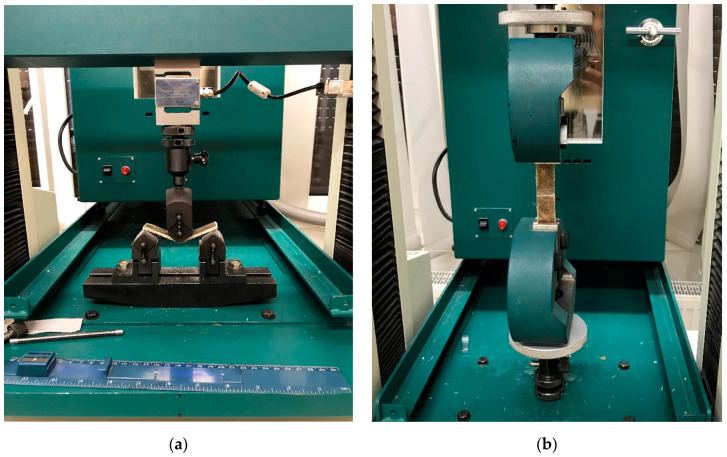
Three-point bending test for flexural properties on Tinius Olsen (**a**); interlaminar shear strength test on Tinus Olsen (**b**).

The flexural tests were performed to find the mechanical properties of the composite materials. This test is used to calculate the maximum stress and strain for the addition of external load. The specimens were tested on a Tinius Olsen H10K machine (Horsham, PA, USA) at a constant strain rate of 0.10 mm/min and speed as 20 mm/min. The force accuracy as 0.5% of applied load and 0.001 mm/min speed resolution. All the samples were tested at room temperature, and every time, five specimens were tested for each composite to get the average values. The specimens were tested as per ASTM D 790 standards. The dimensions of the samples are taken as 100 mm total length, 80 mm span length, 20 mm, and 5 mm thickness. The samples were allowed for three-point bending tests. The apparatus used for the flexural test is shown in Figure 4a. The strength of the composites was calculated by using the following relation.

(1) $$\sigma_{f}=\frac{3PL}{2bd^{2}}\;\mathrm{MPa}$$

where $\sigma_{f}$ is flexural strength, *P* is maximum load, *L* is length of the composites, *b* is width, and *d* is thickness.

### 3.2. Flexural Modulus Test

The flexural modulus of composite material is defined as the ability for that composite to deform. It is calculated from the slope of the stress and displacement curve. It is also called a tangent modulus and modulus of elasticity. The following relation was used to find out the flexural modulus.

(2) $$E_{B}=\frac{L^{3}m}{4bd^{3}}\;\mathrm{MPa}$$

where $E_{B\;}$ is flexural modulus, *L* is length, *m* is slope of stress-strain curve, *b* is width, and *d* is thickness.

### 3.3. Interlaminar Shear Strength

Shear tests of various types are widely used in the polymers industry to find the strength of the reinforcement to matrix materials. The interlaminar shear strength is the failure shear occurred in a composite material when the transverse load applied to it. The samples were taken for a notch cut near to the center on both sides of the specimen before going for the testing. All the specimens were allowed for testing on the Tinius Olsen H10K apparatus. The specimens were tested as per ASTM D-3846-02 standards. The dimensions of the samples are taken as 100 mm total length, 79.6 mm span length, 12.7 mm width, 5 mm thickness, 2.5 mm notch depth, and 6.4 mm distance between the notches. The following relation was used to calculate the shear strength of the composites. The apparatus used for this test is shown in Figure 4b.

(3) $$\sigma_{S}=\frac{P}{A}\;\mathrm{MPa}$$

where *P* is maximum load and *A* is area.

### 3.4. Moisture Absorption Test

This test is used to find out the rate of water (moisture) absorbed by the composite when it is exposed to the wet medium. To evaluate the absorption rate, the composite sample is placed in a container full of distilled water. These samples were placed in an oven for 20 min at 60 °C for the heat treatment process, before placing it in the container to eliminate the moisture in it. The specimens were tested as per ASTM D-570. The dimensions of the samples are taken as 76 mm length, 5 mm thickness, and 20 mm width. The % of weight gain by the composites were calculated in successive time intervals for five days. The following relation was used to calculate the moisture absorption of composite samples.

(4) $$\%\;Weight\;gain=\frac{Final\;weight-Initial\;weight}{Initial\;weight}\times 100$$

### 3.5. Contact Angle Measurement Test

**Figure 5:**
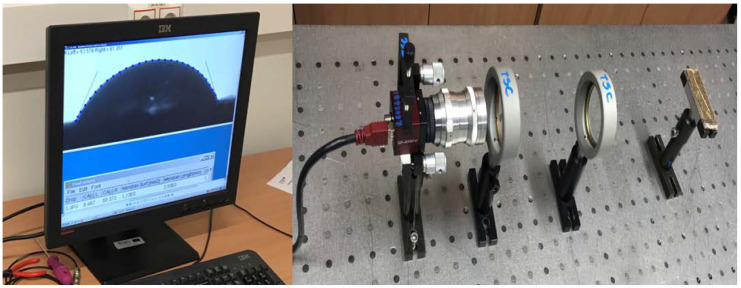
Contact angle measurement apparatus.

The contact angle measurement technique is used to find out the wettability of the composite surfaces. The wetting property defines the ability of the liquid constituent to hold the interaction when it contacts the solid surface. It was found that the contact angle is an attractive method to observe the behavior of the liquid on a composite surface. It is used to find the hydrophobicity of the composite surfaces. The specimens were tested as per ASTM D-7334. The dimensions of the specimens are taken as 80 mm length, 20 mm width, and 5 mm thickness. A total of five samples were tested for each composite. In this method, a water droplet is placed on the composite surface with the help of a glass pipet. The volume of the water droplet kept constant for all the composites. The experimental setup used for contact angle measurement is shown in Figure 5, and it consists of an image processor, a moveable holder, camera, optical lenses, test sample holder, and a composite placed on a holder rest.

## 4. Results and Discussions

### 4.1. Flexural Properties

The analytical results for banana-pineapple and hemp-flax fabricated hybrid composites, obtained from flexural properties testing, are mentioned in the following Table 3.

**Figure 6:**
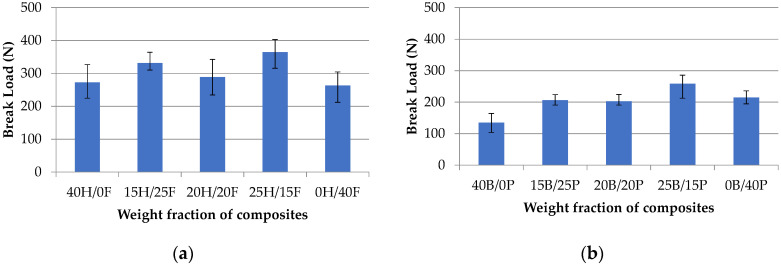
Break load vs. weight fraction of hemp/flax (H/)F composites (**a**) and banana/pineapple (B/P) composites (**b**).

Figure 6a,b show the relation between break load and weight fraction of various composites. In the first set of composites pure hemp (40H/0F) and pure flax (0H/40F) composites showed the least break load and hybrid composites showed improved break load. Additionally, 25H/15F showed the highest break load as 364.9 N at the breakpoint of the composite. In the second set of composites pure banana (40B/0P) and 20B/20P composites exhibited low break load as 135 N and 202.6 N, whereas 25B/15P showed the highest break load as 258.7 N. Hemp and flax fiber hybrid composites showed superior properties than banana and pineapple composites.

**Figure 7:**
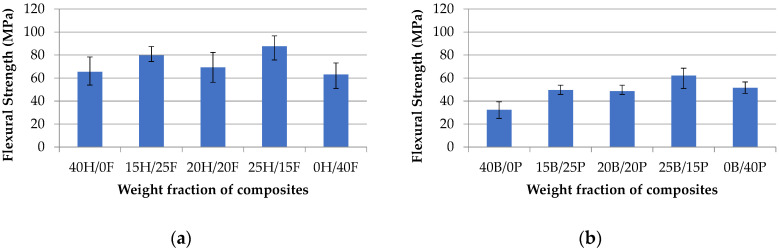
Flexural strength vs. weight fraction of H/F composites (**a**) and B/P composites (**b**).

The flexural properties of various weight fraction of composites are given in Figure 7a,b. Among all the composites, the 25H/15F hybrid composite has the highest flexural strength at 87.57 MPa, and pure banana (40B/0P) has the least strength at 39.36 MPa. Hemp and flax fiber composites exhibited a range between 63 and 88 MPa, whereas banana and pineapple composites showed in between 38 to 69 MPa. This is due to: (1) the hybridization impact for the fibers contributing superior flexural strength to the composites in both the cases; (2) the poor bonding between fiber reinforcement and matrix material in the pure composites leading to the agglomeration, hence, the loss, of strength, and fabrication errors might be the reason for lowering the strength in pure composites. The obtained results are proved that the hybridization of natural fibers gives superior results when compared to the individual fiber composites and natural fibers combined with synthetic fibers. It was proved from the previous results, giridharan worked on preparation and property evaluation of glass/ramie fibers reinforced epoxy hybrid composites [32]. The results were indicated that the glass and ramie hybrid composites have the flexural strength properties range of 50 and 65 MPa, which is less than hemp and flax hybrid composites. Singh et al. [33] investigated the tensile and flexural properties of hemp fiber reinforced virgin-recycled HDPE matrix composites. Hemp fiber composites were fabricated by varying the weight fraction of fiber and matrix material. The results showed that the hemp fiber composites have flexural properties range from 16 to 23 MPa, which is far less than the hemp/flax and banana/pineapple hybrid composites. Bazan et al. [34] investigated the mechanical, thermal, and ageing properties of bio-based polyethylene natural fiber composites. The results indicated that flexural properties were increased by increasing the fiber content and a similar trend followed up to 40% of fiber fraction and then decreased. It was noted that the properties are varied between 20 and 45 MPa. Sinha et al. [35] investigated the mechanical properties of natural fiber polymer composites. The results stated that coconut fiber epoxy composites showed flexural strength as 64.6 MPa. Raghuram et al. [36] worked on characteristics of treated natural fiber epoxy composites with time-variant. The result stated that natural fiber epoxy-based composites showed flexural strength values between 33.35 and 70 MPa, which is better than the glass fiber blend results. Kumar [37] worked on a dataset on mechanical properties of natural fiber reinforced polyester composites for engineering applications. It was observed from the results that sisal, coconut coir fiber composites exhibited flexural strength ranging from 26.2 to 40.3 MPa. By considering the previous results, the hybridization of natural fibers has shown a significant impact on mechanical properties.

**Figure 8:**
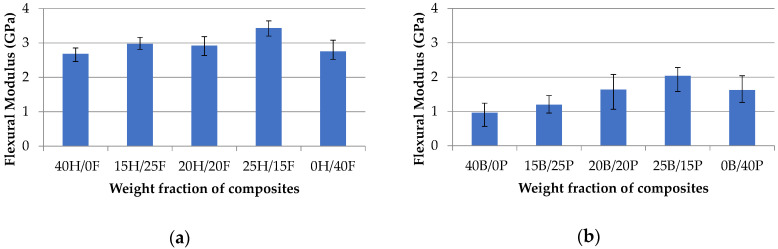
Flexural modulus vs. weight fraction of H/F composites (**a**) and B/P composites (**b**).

Figure 8a,b show the trend of the flexural modulus of various composites. It was observed from the results that banana and pineapple composites showed lower flexural modulus than hemp and flax composites. Pure banana (40B/0P) has the least among all, and the 15H/25F composite has the highest flexural modulus at 2.97 GPa. All hybrid composites showed the flexural modulus close to each other in both the sets.

### 4.2. Interlaminar Shear Strength

The data presented in Table 4 show that the interlaminar shear strength properties for all the composites. The tests were performed to observe the failure shear of the composites. The results were considered when the failure shear occurred in between the notches.

**Figure 9:**
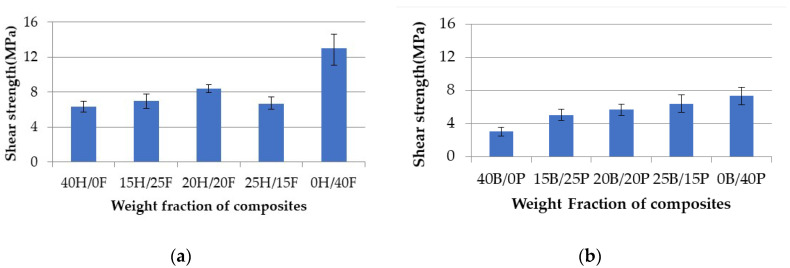
Shear strength vs. weight fraction of H/F composites (**a**) and B/P composites (**b**).

Figure 9a,b show the interlaminar shear strength with respect to the weight fraction of all the composites. In both cases, pure composites showed the highest shear strength values and hybrid composites showed intermediate results. The pure hemp (40H/0F) composite showed the highest shear strength at 13.01 MPa, and the pure banana (40B/0P) composite showed the least shear strength at 3.03 MPa. The hybridization effect is negligible, because there is no such variation in results in both sets.

### 4.3. Moisture Analysis

The amount of water absorbed by a composite material mainly depends on the fiber content, immersion temperature, area of contact to water, and void content. The results were calculated at regular intervals of time for five days.

**Figure 10:**
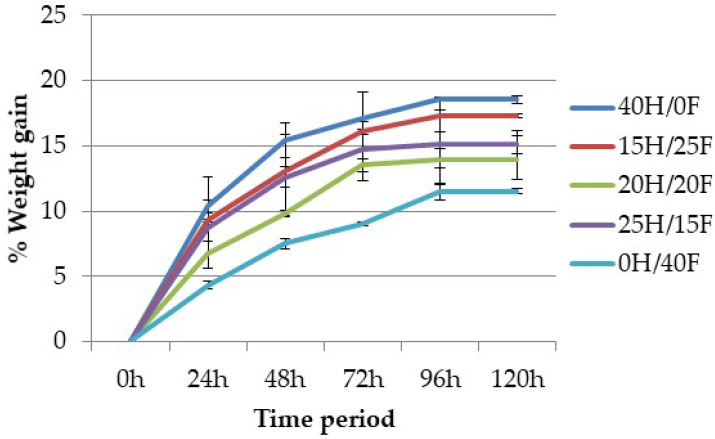
Percentage weight gain vs. time period for hemp and flax composites.

**Figure 11:**
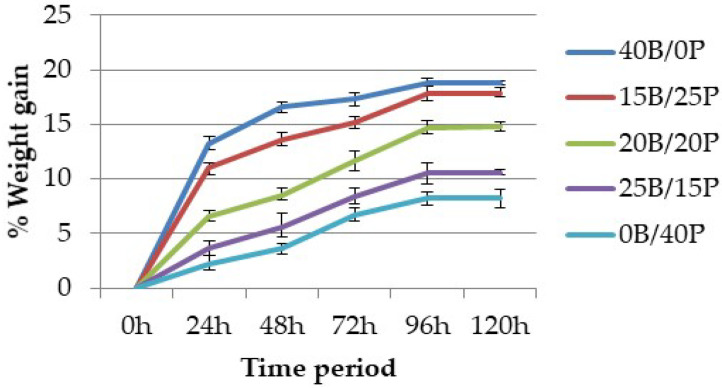
Percentage weight gain vs. time period for banana and pineapple composites.

Figure 10 and Figure 11 show the percentage of weight gain in all fabricated composites. It was observed from the results that the percentage of weight gain in the composite increases with the increase of time. The increase rate remains constant after 96 h of time in both cases, which means that the composite reaches an equilibrium state after 96 h. Pure hemp and pure banana composites showed the highest and pure flax and pure pineapple the lowest values of absorption rate, whereas hybrid composites showed intermediate results. This can be attributed to: (1) the presence of hydroxyl groups in the cellulose structure, which attracts more water and binds the cells through hydrogen bonding; (2) the void content (porosity), which also attracts water uptake in the composite due to the fabrication errors.

### 4.4. Contact Angle Measurement

**Figure 12:**
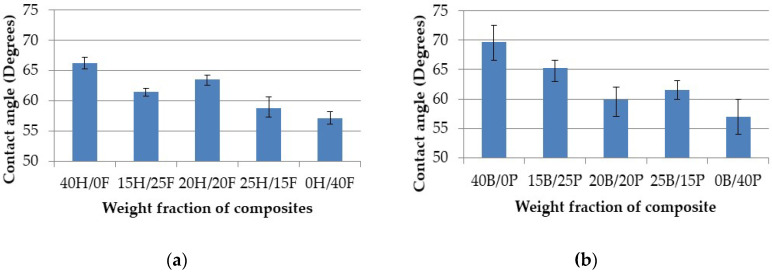
Contact angle vs. weight fraction of H/F composites (**a**) and B/P composites (**b**).

The wettability properties or hydrophilic nature of the composites are calculated by using the contact angle measure analysis. The contact angle was measured with the image processing apparatus as shown in Figure 5. All the values are measured (recorded) in a dark place and for each composite five samples were tested. The contact angle of the composites was measured and with deviation in each value presented in Figure 12a,b.

In general, the highest contact angle is recorded on rough composite surfaces, and the lowest is recorded on smooth composite surfaces. If the contact angle is less than 90°, then the composite has a hydrophilic nature. If it is more than 90°, then the composite has hydrophobic nature. It was found that all the fabricated composites showed a contact angle of lower than 90°, which means that composites have hydrophilic nature. Hemp and flax fiber hybrid composites exhibited a contact angle between 58° to 70°. Banana and pineapple composites showed between 55° and 75°. Hybrid composites showed an intermediate contact angle in both cases, and pure composites showed the highest contact angle and the lowest contact angle. The is due to both banana and pineapple fibers being cellulose rich fibers, and hybrid composites having a smooth surface finish. From the overall results, it was clear that the hemp and flax fiber composites have a smoother surface finish than the banana and pineapple composites.

## 5. Conclusions

Natural fiber reinforced epoxy resin hybrid fiber composites of varying weight fractions were fabricated and characterized. Two sets of composites (H/F and B/P) were fabricated based on the rule of hybridization mixtures, by using the hand lay-up technique for comparison purposes. Hybrid composites showed improved mechanical properties compared to pure composites in both cases. From the mechanical properties testing of composites in the first set (hemp and flax), it was found that the hybrid composites showed higher flexural properties than pure hemp and pure flax composites. The 25H/15F composite showed a flexural strength and flexural modulus at 87.57 MPa and 3.43 Gpa, respectively. The pure flax composite showed the least flexural strength and modulus at 63.10 MPa and 2.75 GPa, respectively. In the second set (banana and pineapple) of composites, it was found that the 25B/15P composite showed the highest flexural strength and modulus at 68.54 MPa and 2.02 GPa, respectively. The 40B/0P composite showed the least flexural strength and modulus at 39.36 MPa and 0.96 GPa, respectively. In both cases, hybrid composites showed the intermediate interlaminar shear strength, and pure composites showed the highest and lowest values. From contact angle measurement analysis, all the composites showed a contact angle at less than 90°, which means composites are exhibiting hydrophilic surface properties. There is no such deviation in the contact angle results among all composites, but pure composites showed the highest contact angle in both cases, which means pure composites have a rough surface finish. From the moisture analysis results, it was found that all the composites absorbed water when it was placed in a water container. The percentage weight gain of the composites remained constant after 96 h in both cases, which means that the composites have reached an equilibrium position. Hybrid composites absorbed less water than pure composites in both cases. By considering the overall results, the hybrid composites showed improved properties compared to pure composites, and hemp and flax fiber composites have superior properties than banana and pineapple composites. Hemp and flax fibers are a potential replacement for reinforcements in the composites. Hemp and flax fibers can be used for structural applications.

## Figures and Tables

**Table 1:** The chemical and mechanical properties of natural fibers [29,30].

Properties	Banana	Pineapple	Hemp	Flax
Density ($g/{\mathrm{cm}}^{3}$)	1.35	1.44	1.48	1.4
Young’s modulus (GPa)	27–32	60.82	70	60–80
Elongation (%)	2.6–5.9	2.2	1.6–4.0	1.2–1.6
Cellulose (%)	63–83	12–60	70–74	64–42
Hemi cellulose (%)	6–19	19.5	21–24	16
Lignin (%)	5–10	4.6–11	3.7–5.7	2–2.2
Moisture (%)	10.71	8–11.8	6.2–12	8–12
Microfibrillar angle (°)	11–12	12–14	2–6	5–10

**Table 2:** Mechanical properties of epoxy resin and hardener [31].

Property	Epoxy Resin	Hardener
Density ($g/{\mathrm{cm}}^{3})$	1.15–1.18	0.97–0.99
Specific gravity	1.14 ± 0.1	1.02 ± 0.1
Color	Colorless	Clear or brown
Viscosity at 25 °C (MPa)	550 ± 50	600
Pot life (min) at 23 °C	30 ± 10	30 ± 10
Mixing proportion	10	2
Gel time (h) at 23 °C	24–36	24–36

**Table 3:** Flexural strength properties.

Composites	Break Load	Flexural Strength (MPa)	Flexural Modulus (GPa)
	(N)		
40H/0F	272.5	65.39	2.68
0H/40F	262.9	63.10	2.75
15H/25F	331.8	79.64	2.97
20H/20F	288.9	69.34	2.92
25H/15F	364.9	87.57	3.43
40B/0P	135.0	39.36	0.96
0B/40P	214.3	46.70	1.62
15B/25P	206.1	53.76	1.19
20B/20P	202.6	45.84	1.63
25B/15P	258.7	68.54	2.02

**Table 4:** Interlaminar shear strength properties.

Composites	Break Load (N)	Interlaminar Shear Strength (MPa)
40H/0F	513.29	6.32
0H/40F	1057.67	13.01
15H/25F	568.15	6.99
20H/20F	682.27	8.39
25H/15F	540.67	6.65
40B/0P	246.30	3.03
0B/40P	594.77	7.32
15B/25P	403.53	4.96
20B/20P	462.42	5.69
25B/15P	518.70	6.38
